# Risk factors of chronic hepatitis in antiretroviral-treated HIV infection, without hepatitis B or C viral infection

**DOI:** 10.1186/1742-6405-10-21

**Published:** 2013-07-26

**Authors:** Thep Chalermchai, Narin Hiransuthikul, Pisit Tangkijvanich, Suteeraporn Pinyakorn, Anchalee Avihingsanon, Jintanat Ananworanich

**Affiliations:** 1 Department of Preventive and Social Medicine, Faculty of Medicine, Chulalongkorn University, Bangkok, Thailand; 2 SEARCH, The Thai Red Cross AIDS Research Center, Bangkok, Thailand; 3 Department of Biochemistry, Faculty of Medicine, Chulalongkorn University, Bangkok, Thailand; 4 HIV-NAT, The Thai Red Cross AIDS Research Center, Bangkok, Thailand; 5 Department of Medicine, Faculty of Medicine, Chulalongkorn University, Bangkok, Thailand

**Keywords:** Chronic hepatitis, HIV infection, Risk factors

## Abstract

**Background:**

Increasing rates of non-AIDS defining illnesses, and in particular liver diseases, have been found after the initiation of highly active antiretroviral therapy. However, there is little evidence concerning the risk factors for and clinical characteristics of liver disease in antiretroviral (ARV)-treated HIV infection, in the absence of hepatitis B or C viral co-infection.

**Methods:**

A nested case–control study of HIV infected volunteers, matched by starting date of anti-retroviral treatment, was conducted in a Thai cohort studied from Nov 2002 - July 2012. Cases were defined as those subjects with an elevated alanine aminotransferase (ALT ≥ 40 IU/L) at two consecutive visits six months apart, while controls were defined as individuals who never demonstrated two consecutive elevated ALT results and had a normal ALT result (< 40 IU/L) at their last visit. Both groups had normal ALT levels prior to ARV initiation. Clinical demographics and risk factors for chronic hepatitis including HIV-related illness, ARV treatment and metabolic diseases were collected and analyzed. Conditional logistic regression was used to determine risk factors for chronic hepatitis in HIV infection.

**Results:**

A total of 124 matched pairs with HIV infection were followed over 3,195 person-years. The mean age (±SD) was 33.0 ± 7.3 years, with 41.1% of subjects being male. The incidence of chronic hepatitis was 5.4 per 100 person-years. The median time from initiation of ARV to chronic hepatitis was 1.3 years (IQR, 0.5-3.5). From univariate analysis; male sex, plasma HIV-1 RNA level > 5 log _10_ copies/ml, metabolic syndrome at baseline visit, high BMI > 23 kg/m^2^, abnormal HDL cholesterol at time of ALT elevation and treatment experience with NNRTI plus boosted PI were selected (p value < 0.2) to the final model of multivariate analysis. Male sex had 3.1 times greater risk of chronic hepatitis than the females by multivariate analysis (adjusted OR, 95% CI: 3.1, 1.5-6.3, p =0.002). High BMI ≥ 23 kg/m^2^ was also associated with 2.4 times greater risk of chronic hepatitis (adjusted OR, 95% CI: 2.4, 1.2-4.8, p = 0.01).

**Conclusions:**

Chronic hepatitis in ARV-treated HIV-infected patients is common and may lead to a major health care problem. Male sex and high BMI ≥ 23 kg/m^2^ carry higher risks for developing chronic hepatitis in this study. Therefore, these patients should be closely monitored for long-term hepatotoxicity.

## Background

Human immunodeficiency virus (HIV) infection continues to be a major global health problem. An estimated 33.4 million people are currently living with HIV worldwide [1]. The era of highly active antiretroviral therapy (HAART) for the treatment of HIV-infected individuals has led to a dramatic reduction in AIDS-defining illnesses and mortality [2]. However, an inverse trend has also been observed with increasing rates of non-AIDS defining illnesses including liver diseases manifesting themselves in the context of longer life expectancy [3,4]. Little is known about the risk factors for and clinical characteristics of liver disease in Asian HIV infection. Manifestations of liver disease in HIV infection include mild, asymptomatic rises in liver transaminase levels, chronic steatohepatitis, liver cirrhosis and more severe forms resulting in hepatic failure. In general, chronic liver disease in HIV mono-infected patients usually present with no or only mild symptoms and rising serum transaminase levels [5]. Research into the HIV-infected subgroup with mildly elevated transaminase levels is so far minimal. Previous studies suggested that the majority of mild, asymptomatic chronic hepatitis in HIV infection were undiagnosed [5]. Failure or delay in the diagnosis and treatment of liver disease in HIV infection may result in long-term liver morbidities such as chronic steatohepatitis, liver cirrhosis or hepatocellular carcinoma and, importantly, may also increase the rate of liver-related mortality [6].

The etiologies of liver disease seen in HIV infection include hepatitis B or C virus co-infection, medication-induced hepatitis from antiretroviral (ARV) or non-ARV drugs (such as anti-tuberculous and anti-lipemic agents), metabolic syndrome, excessive alcohol consumption and HIV infection itself [3].

ARV medications that commonly result in rising transaminase in HIV infection include stavudine (d4T), nevirapine (NVP) and high dose ritonavir (RTV) [7-9]. Other reported risk factors were being men who has sex with men (MSM), and having a history of arterial hypertension or a body mass index (BMI) greater than 25 kg/m^2^[9-11].

Chronic hepatitis in HIV infection has also been associated with poor HIV-specific treatment outcomes, such as current CD4+ T-lymphocyte counts < 200 cells/mm^3^ and detectable HIV-1 RNA levels [11].

Previous studies of chronic hepatitis in HIV infection were mostly cross-sectional. Most of the studies were conducted in developed countries. There is a lack of clinical evidence in developing countries especially in patients treated with ARV. Results from this study will support healthcare personnel in understanding and selecting appropriate treatment for a resource-limited setting.

This study aims to determine clinical demographic and the risk factors of chronic hepatitis in ARV-treated, HIV-infected Thai patients without hepatitis B or C co-infection who were followed longitudinally for up to 10 years.

## Materials and methods

We conducted a nested case–control study utilizing data from a long term observational study (Clinical trial.gov, NCT 00411983) for HIV-infected individuals who had previously participated in clinical studies and continued into a long term cohort after the completion of the specific study. The study participants were scheduled for follow-up at The HIV Netherlands Australia Thailand Research Collaboration or HIV-NAT clinic in Bangkok, Thailand on a semi-annual basis to monitor their long-term outcomes for AIDS-defining and non-AIDS defining illnesses as well as adverse events from ARV treatment. Medical history, physical examination and history of ARV treatment were recorded. Subjects underwent blood draw for complete blood count (CBC), serum alanine aminotransferase level (ALT) and serum creatinine level. CD4+ T lymphocyte counts and HIV-1 RNA levels were also measured at each study visit.

Our study focused on liver complications in HIV without hepatitis B or C co-infection. The inclusion criteria were HIV-infected individuals, older than 18 years of age, negative hepatitis B surface antigen (HBs Ag) or HBV DNA and negative HCV antibody (anti-HCV) or HCV RNA. Exclusion criteria were patients who did not have a recorded viral hepatitis serology result, had an abnormal ALT level at their baseline visit or prior to ARV treatment (ALT ≥40 IU/L), unavailable baseline ALT level or had less than 12 months of follow up. Written, informed consent was obtained from all enrollees. This specific study was approved by the Ethics Committee of the Faculty of Medicine, Chulalongkorn University, Bangkok, Thailand.

For this case–control study, the cases, or chronic hepatitis was defined as those with elevated ALT ≥ 40 IU/L at 2 consecutive visits 6 months apart after the initiation of ARV [7,8]. The controls was defined as those patients who never had two consecutive ALT ≥ 40 IU/L and still had normal ALT at the last visit. Both groups had normal ALT at pre-ARV initiation (baseline).

The case and control were matched 1:1 for duration from ARV initiation (± 6 months).

All clinical data and laboratory testing for comparison were selected by utilizing data at baseline and at the time chronic hepatitis occurred in the cases and the data at the same period (± 6 months) for the matched controls.

All analytic data for this study were censored on July 1, 2012.

The duration of the study was calculated from the time of initiation of ARV to the last follow-up visit.

The onset of chronic hepatitis was calculated from the time of ARV initiation to the diagnosis of chronic hepatitis.

### Definitions

*HIV infection* was defined by a positive result for HIV-specific antibodies by enzyme-linked immunosorbent assay (ELISA) and/or HIV-1 RNA by the Roche Amplicor HIV-1 Monitor Test v1.5.

*Hepatitis B virus* infection was defined by a positive result for HBsAg using the ARCHITECT HBsAg qualitative assay (ABBOTT Max-Planck-Ring 2, Germany) and/or detectable HBV DNA by polymerase chain reaction (PCR).

*Hepatitis C virus* infection was defined by a positive result for HCV-specific antibodies using the ARCHITECT Anti-HCV assay or detectable HCV RNA by PCR.

*Severity grading of chronic hepatitis* was classified according to the following ranges of ALT level: grade 1, 1.0 – 2.5 times the upper limit of normal (1.0-2.5 × ULN); grade 2, 2.6–5.0 × ULN; grade 3, 5.1–10 × ULN; grade 4, >10 × ULN. Severe hepatitis was defined by at least grade 3 ALT elevation [12].

Severity of HIV infection was classified by Centers for Disease Control and Prevention (CDC) 1993 guidelines [13].

*Body mass index* (BMI) was calculated as weight in kilograms divided by height in meters squared. BMI categories specific for Asian individuals were assigned as follows: BMI <18.5 (underweight), BMI = 18.5 – 22.9 (normal), BMI = 23–24.9 (overweight) and BMI ≥25.0 (obese) [14].

*Dyslipidemia* was defined as serum triglyceride level ≥ 150 mg/dL, HDL-cholesterol ≤ 40 mg/dL for males, ≤ 50 mg/dL for females, LDL-cholesterol ≥ 130 mg/dL and total cholesterol ≥200 mg/dL [15,16].

*Elevated blood pressure* was defined as a systolic blood pressure > 130 mmHg or diastolic blood pressure > 85 mmHg, or known history of hypertension [16].

*Impaired fasting plasma glucose* was defined as a fasting plasma glucose ≥ 100 mg/dL or known history of diabetes mellitus [16].

*Metabolic syndrome* was defined as having at least 3 of 5 criteria serum triglyceride level ≥150 mg/dL, serum HDL-cholesterol ≤40 mg/dL for male or ≤50 mg/dL for females, elevated blood pressure, impaired fasting plasma glucose and BMI ≥ 23 kg/m2 or waist circumference ≥ 90 cm. for male or ≥ 80 cm. for females [14].

*Clinically-diagnosed lipodystrophy* was defined as those with lipoatrophy or lipohypertrophy, truncal obesity or facial lipodystrophy [17].

The main risk factors that were evaluated included clinical characteristics [age, gender, sexual risk behavior, HIV-related illness and disease severity, baseline CD4^+^ T lymphocyte counts, plasma HIV-1 RNA level and metabolic diseases] and clinical parameters and laboratory assessments at an event visit [previous history of ARV treatment, concomitant medications, clinical and laboratory findings of HIV-related illnesses and metabolic diseases].

### Statistical and data analysis

Clinical characteristics were described as frequency and percentage for categological data. Continuous data were reported as mean (x̄) and standard deviation (SD) if normally distributed or as median and inter-quartile range (IQR) if not normally distributed [18]. For inferential data analysis, the McNemar’s test was used for categorical data. For continuous data, the paired student’s t-test was used if the data had normal distribution, while the Wilcoxon signed-rank test was used if they showed non-normal distribution. Factors with p value less than 0.2 from univariate analysis were selected to the model of multivariate analysis. Conditional stepwise, logistic regression was used to evaluate the risk factors of chronic hepatitis. Odds ratio (OR) and adjusted OR with 95% confidence intervals (CI) were reported to demonstrate an association between significant factors with chronic hepatitis. Statistical significance was defined as a p value less than 0.05. We used STATA/LC version 11.2 for Windows.

## Results

**Figure 1:**
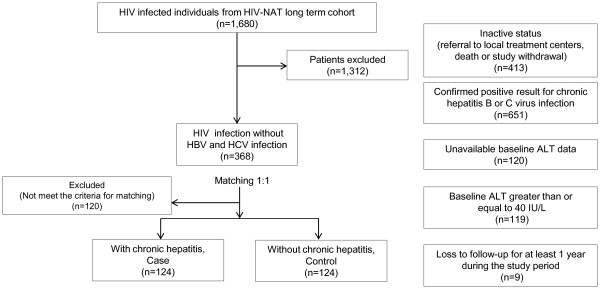
Demonstrate flow of the study.

A total of 1,680 HIV-infected individuals participated in the long term HIV-NAT cohort during the period of November 2002 to July 2012. We excluded individuals from the present study owing to inactive status (referral to local treatment centers, death or study withdrawal) at the time of data analysis (n = 413), a confirmed positive result for chronic hepatitis B or C virus infection (n = 651), unavailable baseline ALT data (n = 120), baseline ALT ≥ 40 IU/L (n = 119) and loss to follow-up for at least 1 year (n = 9) (Figure 1).

### Characteristics of chronic hepatitis in cases

There were 124 study cases. An incidence rate of chronic hepatitis was 5.4 per 100 person-years (Table 1). The mean ALT level at the time of diagnosis of chronic hepatitis was 73 ± 107 IU/L [median ALT level was 52 IU/L, (IQR, 46–65)]. There were 98.4% of these individuals who were initially found to have mild ALT (grade 1 or 2) elevation, while 1.6% (n = 2) were found to have severe hepatitis. The mean (±SD) AST level was slightly higher than the normal value (58 ± 49 IU/L). The median duration from the time of an initiation of ARV to the diagnosis of chronic hepatitis was 1.3 years (IQR, 0.5-3.5). The following symptoms were reported at time of ALT elevation; however, their relatedness to ALT elevation cannot be confirmed: nausea and/or vomiting (24.2%), fatigue (11.3%), anorexia (7.3%), flatulence (4.0%) and others (4.8%). All were grades 1 or 2. No abnormal severe symptoms, liver failure or hepatic encephalopathy was reported.

**Table 1:** Characteristics of chronic hepatitis in cases of HIV patients with chronic non HBV/HCV hepatitis*

**Clinical parameters**	**Case(n =124 )**
Incidence rate , per 100 person-years	5.4
Median (IQR), duration from starting ARV to hepatitis, years	1.3(0.5–3.5)
Mean ALT level at time of ALT elevation, IU/L (±SD)	73 ± 107
Mean AST level at time of ALT elevation, IU/L (±SD)	58 ± 49
Mean alkaline phosphatase level at time of ALT elevation, IU/L (±SD)	97 ± 54
Severity grading of chronic hepatitis **, n (%)	
o Mild (grade 1 or 2)	122 (98.4)
o Severe, at least grade 3	2 (1.6)
Symptoms at time of ALT elevation, n (%)	
o Any	64(51.6)
o Nausea/ vomiting	30(24.2)
o Fatigue	14(11.3)
o Anorexia	9(7.3)
o Flatulence	5(4.0)
o Weight loss	4(3.2)
o Jaundice	1(0.8)
o Liver tenderness	1(0.8)

*Abbreviation: HIV* Human immunodeficiency virus, *HBV* Hepatitis B virus, *HCV* Hepatitis C virus, *ALT* Alanine aminotransferase, *AST* Aspartate aminotransferase, *SD* Standard deviation.

* Chronic hepatitis was defined as those with elevated ALT ≥40 IU/L at 2 consecutive visits 6 months apart.

**Severity grading of chronic hepatitis was classified by using ALT level: grade 1, 1.0 – 2.5 times the upper limit of normal (1.0-2.5 × ULN); grade 2, 2.6–5.0 × ULN; grade 3, 5.1–10 × ULN; grade 4, >10 × ULN [12]. Severe hepatitis was defined as having at least grade 3 ALT level.

### Baseline demographics

From a total of 124 HIV-infected, matched pairs by the date of starting ARV treatment were selected. Male gender was more commonly found in the cases than the controls. The mean ages in the cases and the controls were similar. The proportion of men who have sex with men (MSM) were slightly higher in the cases than the controls with borderline significance (p = 0.08) (Table 2).

**Table 2:** Clinical characteristics of HIV patients with or without chronic non HBV/HCV hepatitis at baseline visit**

**Clinical parameters**	**Case**	**Control**	**p value**
	**(n = 124)**	**(n = 124)**	
Male sex (n,%)	65(52.4)	37(29.8)	0.001*
Mean age (years), ± SD	32.7 ± 7.4	33.3 ± 7.2	0.56
o ≤ 25 (n,%)	16(12.9)	12(9.7)	0.85
o 25–34.9 (n,%)	68(54.8)	70(56.4)	
o 35–44.9 (n,%)	31(25.0)	34(27.4)	
o ≥ 45 (n,%)	9(7.3)	8(6.5)	
BMI ≥ 23 kg/m^2^ (n,%)	39(31.4)	27(21.8)	0.09
Elevated blood pressure/ hypertension (n,%)	18(14.5)	18(14.5)	1.00
Metabolic syndrome (n,%)	9(7.3)	3(2.4)	0.08
Sexual risk behavior (n,%)			0.08
o Heterosexual	83(66.9)	98(79.0)	
o MSM	37(29.9)	23(18.6)	
o Others	4(3.2)	3(2.4)	
CDC classification (n,%)			0.21
o Category A or B	110(88.7)	117(94.4)	
o Category C	14(11.3)	7(5.6)	
Laboratory measurement (n,%)			
o CD4+ cell count ≤ 200 cells/mm3	53(42.7)	50(40.3)	0.66
o Nadir CD4+ cell count ≤ 200 cells/mm3	67(54)	67(54)	1.00
o HIV-1 RNA level ≥ 5 log 10 copies/ml	41(33.1)	31(25)	0.13
o Impaired fasting plasma glucose ≥ 100 mg/dL	21(16.9)	16(12.9)	0.87
o Total cholesterol ≥ 200 mg/dL	25(22.7)	27(24.1)	0.87
o Triglycerides ≥ 150 mg/dL	25(22.7)	26(23.2)	0.88
o HDL cholesterol ≤ 40 mg/dL (male) or ≤ 50 mg/dL (female)	55(51.4)	57(52.3)	0.70
o LDL cholesterol ≥ 130 mg/dL	2(1.8)	1(0.9)	0.57
Median, duration of study (years) (IQR)	9.6(6.7–11.9)	9.6(6.7–11.9)	0.97

*Abbreviation: HIV* Human immunodeficiency virus, *HBV* Hepatitis B virus, *HCV* Hepatitis C virus, *SD* Standard deviation, *BMI* Body mass index, *MSM* Men who have sex with men, *CDC* The centers for disease control and prevention, *HDL Cholesterol* High density lipoprotein cholesterol, *LDL Cholesterol* Low density lipoprotein cholesterol and *IQR* Interquartile range.

*Significant value with p value less than 0.05.

**Chronic hepatitis was defined as those with elevated ALT ≥ 40 IU/L at 2 consecutive visits 6 months apart.

Advanced, HIV-related illnesses were found in 11.3% and 5.6% of the case and control groups respectively. There was no significant difference in the proportions of CD4+ cell count less than 200 cells/mm3 and HIV-1 RNA level greater than 5 log _10_ copies/ml between the two groups.

The proportion of individuals with BMI greater than 23 kg/m^2^ was slightly higher in the cases than the controls but did not reach statistical significant difference (p = 0.09). There were only 12 patients who met the criteria for metabolic syndrome because of missing data for lipid profiles and waist circumference. There was no significant difference of metabolic syndrome between groups.

There was no difference in the prevalence of elevated blood pressure and hypertension, impaired fasting plasma glucose and lipid profiles between the groups.

### Clinical characteristics at the time of chronic hepatitis (at event visit)

The proportion of individuals with BMI greater than 23 kg/m^2^ was significantly higher in the cases (44.4%) than the controls (20.9%, p = 0.001) (Table 3).

**Table 3:** Clinical characteristics of HIV patients with or without chronic non HBV/HCV hepatitis at time of ALT elevation**

**Clinical parameters**	**Case**	**Control**	**p value**
	**(n = 124)**	**(n = 124)**	
BMI ≥ 23 kg/m 2 (n,%)	55(44.4)	26(20.9)	0.001*
Elevated blood pressure/ hypertension (n, %)	24(19.3)	18(14.5)	0.32
Metabolic syndrome (n, %)	3(4.3)	3(3.0)	0.66
Clinically-diagnosed lipodystrophy	14(11.3)	10(8.1)	0.32
Laboratory measurement (n, %)			
o CD4+ cell count ≤ 200 cells/mm3	15(12.1)	10(8.1)	0.30
o HIV-1 RNA level ≥ 400 copies/ml	11(8.8)	16(9.6)	0.21
o Impaired fasting plasma glucose ≥ 100 mg/dL	15(14.1)	10(9.6)	0.21
o Total cholesterol ≥ 200 mg/dL	56(50.4)	48(42.8)	0.49
o Triglycerides ≥ 150 mg/dL	40(36.0)	31(27.7)	0.29
o HDL cholesterol ≤ 40 mg/dL (male) or ≤ 50 mg/dL (female)	36(34.3)	22(21.6)	0.09
o LDL cholesterol ≥ 130 mg/dL	36(34.3)	25(24.5)	0.32

*Abbreviation: HIV* Human immunodeficiency virus, *HBV* Hepatitis B virus, *HCV* Hepatitis C virus, *BMI* Body mass index, *HDL* High density lipoprotein and *LDL* Low density lipoprotein.

*Significant value with p value less than 0.05.

**Chronic hepatitis was defined as those with elevated ALT ≥ 40 IU/L at 2 consecutive visits 6 months apart.

There was no difference in the proportion with individuals of CD4+ cell count less than 200 cells/mm3, plasma HIV-1 RNA level greater than 400 copies/ml, elevated blood pressure and hypertension, impaired fasting plasma glucose and metabolic syndrome between the groups. The cases tended to have lower HDL than the controls (p = 0.09).

### Anti-retroviral regimens

All patients were treated with ARV (Table 4). The combination of dual nucleoside reverse transcriptase inhibitors (NRTI) with PI (protease inhibitor) (48.4%) or non-nucleoside reverse transcriptase inhibitors (NNRTI) (37.1%) were the most common first regimens. For triple NRTI, the most common regimen was zidovudine (ZDV) combined with lamivudine (3TC) plus didanosine (ddI) (33%). For dual NRTI plus NNRTI, the most common regimens were efavirenz (EFV) combined with tenofovir (TDF) plus 3TC (35.7%) and NVP combined with 3TC and d4T (17.3%). For dual NRTI plus boosted PI, the most common regimens were saquinavir/ritonavir combined with 3TC plus TDF (24.4%) and lopinavir/ritonavir combined with 3TC plus TDF (12.2%). For NNRTI plus boosted PI, the most common regimen was indinavir/ritonavir combined with EFV (44.4%).

**Table 4:** Antiretroviral use* of HIV patients with or without chronic non HBV/HCV hepatitis**

**Clinical parameters**	**Case**	**Control**	**p value**
	**(n = 124)**	**(n = 124)**	
Median, duration of ARV exposure (years) (IQR)	2.2(0.9-5.2)	1.6(0.9-4.4)	0.18
Antiretroviral regimens (n, %)			
o Triple NRTIs	18(14.5)	-	NA
o Dual NRTIs plus NNRTI	46(37.1)	52(41.9)	0.37
o Dual NRTIs plus boosted PIs	58(46.8)	65(54.4)	0.29
o NNRTIs plus boosted PIs	2(1.6)	7(5.7)	0.12

*Abbreviation: NRTIs* Nucleoside reverse transcriptase inhibitors, *NNRTI* Non-nucleoside reverse transcriptase inhibitor, *PIs* Protease inhibitors, *ARV* Antiretroviral, *IQR* Interquartile range, *NA* Non applicable.

* Ever exposed to antiretroviral medication.

**Chronic hepatitis was defined as those with elevated ALT ≥ 40 IU/L at 2 consecutive visits 6 months apart.

Eleven patients developed early acute adverse events from ARV treatment such as severe hepatitis, drug hypersensitivity reaction and severe fatigue. ARV was discontinued and the hepatitis completely resolved. The new regimens for these patients were used as the first ARV regimen for our analysis.

### Concomitant medication

The concomitant medications were similar between the 2 groups (Table 5).

**Table 5:** Concomitant medication use* of HIV patients with or without chronic non HBV/HCV hepatitis**

**Concomitant medications**	**Case**	**Control**	**p value**
	**(n = 124)**	**(n = 124)**	
o Isoniazid	17(13.7)	22(17.7)	0.36
o Pyrazinamide	7(5.6)	6(4.8)	0.78
o Gemfibrosil	7(5.6)	7(5.6)	1.0
o Fenofibrate	11(8.8)	8(6.5)	0.47
o Simvastatin	7(5.6)	5(4.0)	0.53
o Cotrimoxazole	50(40.3)	50(40.3)	1.0
o Dapsone	4(3.2)	3(2.4)	0.71
o Fluconazole	19(15.3)	14(11.3)	0.30
o Analgesic	14(11.3)	17(13.7)	0.33

Footnote:

* Ever exposed to any concomitant medications.

** Chronic hepatitis was defined as those with elevated ALT ≥ 40 IU/L at 2 consecutive visits 6 months apart.

### Risk factor variables for chronic hepatitis

From the univariate analysis; male sex, plasma HIV-1 RNA level > 5 log _10_ copies/ml and metabolic syndrome at baseline visit, high BMI ≥ 23 kg/m^2^ and abnormal HDL cholesterol at time of ALT elevation and treatment experience with NNRTI plus boosted PI were selected to the final model of multivariate analysis (Table 6).

**Table 6:** Univariate and multivariate analysis to evaluate each variable and medication for the risk of chronic non HBV/HCV hepatitis***

**Clinical parameters and ARV**	**Univariate**	p value*	**Multivariate**	p value**
	**OR (95% CI)**		**AOR (95% CI)**	
Male sex	3.0(1.6–5.5)	<0.001	3.1(1.5–6.3)	0.002*
BMI ≥ 23 kg/m2 at time of ALT elevation	3.1(1.7–5.6)	<0.001	2.4(1.2–4.8)	0.01*
Metabolic syndrome at baseline visit	4.0(0.8–18.8)	0.08		
Plasma HIV-1 RNA level ≥ 5 log 10 copies/ml at baseline visit	1.6(0.9–3.0)	0.13		
HDL cholesterol ≤ 40 mg/dL (male) or ≤ 50 mg/dL (female) at time of ALT elevation	1.8(0.9–3.7)	0.09		
Exposure to NNRTIs + boosted PIs regimens	0.3(0.1–1.4)	0.12		

*Abbreviation: OR* Odds ratio, *AOR* Adjusted odds ratio, *BMI* Body mass index, *HDL* High density lipoprotein, *NNRTI* Non-nucleoside reverse transcriptase inhibitor, *PIs* Protease inhibitors,

*P value less than 0.20 from univariate analysis to the model for multivariable analysis **Significant value with p value less than 0.05.

*** Chronic hepatitis was defined as those with elevated ALT ≥ 40 IU/L at 2 consecutive visits 6 months apart.

From the multivariate analysis, male sex (AOR, 95% CI; 3.1, 1.5-6.3, p =0.002) and high BMI ≥ 23 kg/m^2^ (AOR, 95% CI; 2.4, 1.2-4.8, p = 0.01) were the independent risk factors for chronic hepatitis.

## Discussion

Our study examined clinical characteristics and the risk factors of chronic hepatitis by evaluating ALT level as a surrogate marker. We reported a case–control study of 124 matched pairs, ARV-treated HIV-infected individuals without HBV or HCV infection with a follow-up time of 3,195 person-years. Our incidence rate of hepatitis was slightly higher (5.4 cases per 100 person-years) than previously reported in Kovari et al. (3.9 cases per 100 person-years) [9].

The ALT elevation in our study was mostly mild (grade 1–2, 98.4%). There was no report of serious liver disease or hepatic encephalopathy, however, its long-term deleterious consequence is not well defined. The majority of previous studies mainly reported patients with severe elevation of ALT (≥ 5 times, UNL) who also tended to have symptomatic disease [19]. In general, chronic liver disease in HIV usually presents with no or mild symptoms with rising serum transaminase levels. Because such individuals are mostly asymptomatic, delayed diagnosis is common [5,20]. Delayed diagnosis and treatment may lead to long term consequences from chronic hepatitis such as liver fibrosis, resulting in cirrhosis and importantly liver cancer [21,22].

We found that the median time from ARV initiation to chronic hepatitis was 1.3 years (IQR, 0.5-3.5) which was similar to previous report of 1.2 year [9]. As our definition required two consecutive abnormal ALT 6-month apart, this suggests that early detection of chronic hepatitis will require monitoring of symptoms and liver enzymes within the first year of ARV initiation.

Our study confirmed that males had 3.1 times (95% CI, 1.5-6.3) greater risk of chronic hepatitis than females. This finding supports the report by Guaraldi G et al. that non-alcoholic fatty liver disease (NAFLD) was associated with male sex and elevated ALT level [23]. Previous study in HIV-negative US adolescents also supported an association between NAFLD and male sex [24].

Our study concurred that high BMI greater than 23 kg / m^2^ was associated with 2.4 times (95% CI, 1.2-4.8) greater risk of chronic hepatitis. Previous studies showed that high BMI (>25 kg / m^2^) and overt obesity were associated with chronic hepatitis [5,9,11]. However, this study did not show an effect of other metabolic components, likely due to missing information on lipid profiles and waist circumference to diagnose the metabolic syndrome. The metabolic syndrome and high BMI can directly cause insulin resistance and impaired fatty acid oxidation in hepatocytes and subsequently lead to hepatic cellular injury [25,26].

Our findings confirmed the study from the Swiss HIV cohort that HIV-related parameters such as baseline CD4+ cell count and HIV-1 RNA level are not associated with chronic hepatitis. Our finding disagrees with the study of Sterling et al. which reported that detectable HIV-1 RNA level was associated with chronic, rising ALT enzymes. However, that study was a cross-sectional study. Plasma HIV-1 RNA and ALT level were tested only one visit during the study period [11].

We did not find exposure to ARV or concomitant medication to demonstrate an association with chronic hepatitis in contrast to previous reports [7,10]. The Swiss cohort reported that chronic elevated ALT levels were associated with stavudine use of at least 2 years duration but this study examined only a history of any ARV exposure, regardless of the duration of that exposure [9].

The strengths of this study include the availability of prospective longitudinal data collection of ALT that has been shown to accurately reflect liver steatosis. The determination of chronic hepatitis by using ALT level as a surrogate laboratory test increases the generalizability of the data to other resource-limited settings. This test is inexpensive, widely available and easy to perform. However, liver injury may exist in the absence of transaminases elevation. Limitations of this study include a lack of histological and clinical imaging data to confirm liver injury as well as missing data on alcohol use and parameters to diagnose metabolic syndrome. In addition, lipodystrophy may be under diagnosed or under reported. This study did not exclude other causes of chronic hepatitis including metabolic diseases such as hemochromatosis, Wilson’s disease and autoimmune hepatitis and infectious causes including cytomegalovirus or Epstein-Barr virus, chronic hepatitis E virus. Finally, this study also did not examine an association between duration of ARV treatment and chronic hepatitis because it had already matched for the date of ARV initiation.

## Conclusion

In conclusion, chronic hepatitis in ARV-treated HIV infected patients is common and may lead to a major health care problem. Male sex and high BMI ≥ 23 kg/m^2^ carry higher risks for developing chronic hepatitis in this study. Therefore, these patients should be closely monitored for long-term hepatotoxicity.

## Abbreviations

AOR: Adjusted odds ratio; BMI: Body mass index; CDC: Centers for disease control and prevention; HBV: Hepatitis B virus; HCV: Hepatitis C virus; HDL cholesterol: High density lipoprotein cholesterol; HIV: Human immunodeficiency virus; IQR: Interquartile range; LDL cholesterol: Low density lipoprotein cholesterol; MSM: Men who have sex with men; NNRTI: Non-nucleoside reverse transcriptase inhibitor; NRTI: Nucleoside reverse transcriptase inhibitors; OR: Odds ratio; PI: Protease inhibitors; SD: Standard deviation; ULN: Upper limit of normal.

## Competing interests

The authors declare that they have no competing interests.

## Authors’ contributions

NH, PT, AA, JA, TC and SP designed and conducted the study, interpreted the data, and prepared and edited the manuscript. All authors read and approved the final manuscript.
